# RIG-I and IL-6 are negative-feedback regulators of STING induced by double-stranded DNA

**DOI:** 10.1371/journal.pone.0182961

**Published:** 2017-08-14

**Authors:** Xueling Wu, Jun Yang, Tao Na, Kehua Zhang, Andrew M. Davidoff, Bao-Zhu Yuan, Youchun Wang

**Affiliations:** 1 Graduate School of Peking Union Medical College, Beijing, China; 2 Cell Collection and Research Center, National Institutes for Food and Drug Control, Beijing, China; 3 Department of Surgery, St Jude Children’s Research Hospital, Memphis, Tennessee, United States of America; 4 HIV and Sexual Transmitted Viral Vaccine, National Institutes for Food and Drug Control, Beijing, China; University of Tennessee Health Science Center, UNITED STATES

## Abstract

The stimulator of interferon genes (STING) protein has emerged as a critical signal transduction molecule in the innate immune response. Sustained activation of the STING signaling induced by cytosolic DNA has been considered to be the cause of a variety of autoimmune diseases characterized by uncontrolled inflammation. Therefore, it is important to understand the molecular basis of the regulation of STING signaling pathway. Here we demonstrate that the STING protein undergoes a proteasome-mediated degradation in human diploid cell (HDC) lines including MRC-5 following the transfection of double-stranded DNA (dsDNA). The degradation of STING is accompanied by the increased expression of both RIG-I and IL-6. Employing the RIG-I siRNA knockdown and an IL-6 neutralizing antibody greatly inhibits the degradation of STING induced by dsDNA. We further demonstrate that both IL-6 and RIG-I are downstream molecules of STING along the DNA sensor pathway. Therefore, STING degradation mediated by RIG-I and IL-6 may serve as a negative feedback mechanism to limit the uncontrolled innate immune response induced by dsDNA. We have further shown that RIG-I and IL-6 promote STING degradation by activating/dephosphorylating UNC-51-like kinase (ULK1). Interestingly, the STING protein is not significantly affected by dsDNA in non-HDC HEK293 cells. Our study thus has identified a novel signaling pathway for regulating STING in HDCs.

## Introduction

The innate immune system is the first line of defense against disease-causing pathogens and can be triggered by cytosolic DNA derived from the genomes of viruses and bacteria, which acts as a potent activator of the innate immune response. Over the last few years, the molecular basis of DNA sensing by the innate immune system has begun to be understood. It has been demonstrated that a molecule in endoplasmic reticulum, referred to as STING (stimulator of interferon genes), plays a critical role in the production of type I interferons (IFN) induced by the cytosolic DNA [1–4]. STING can be activated through interacting with cytosolic DNA sensors including DNA-dependent activator of IFN-regulatory factors (DAI) [5], IFN-γ-inducible protein 16 (IFI16) [6] and DEAD (Asp- Glu-Ala-Asp) box polypeptide 41 (DDX41) [7]. Meanwhile, STING can also be activated by cyclic dinucleotides generated by Cyclic GMP-AMP synthase (cGAS) [8–10], a cytosolic DNA sensor that binds to microbial DNA as well as self-DNA that invades the cytoplasm. After activation, the STING protein transduces signals to TANK-binding kinase 1 (TBK1) and the transcription factor interferon regulatory factor 3 (IRF3), resulting in the production of type I IFNs to exert antiviral and antibacterial activities [2, 11]. In addition to the production of type I IFN, STING is required for the effective production of some cytokines such as IL-6 and Chemokine (C-C motif) ligand 5 (CCL5) [11], which play important roles in DNA-induced innate immune response.

Retinoic-acid inducible gene I (RIG-I) is a dsRNA helicase enzyme, functioning as a pattern recognition receptor for sensing RNA viruses and being directly associated with mitochondrial antiviral-signaling protein (MAVS) to coordinate downstream activation of TBK1 and IκB kinase epsilon (IKKε) for type I IFN production [12, 13]. Several reports have shown that RIG-I is also a DNA sensor, which is required for evoking type I IFN responses following cytosolic DNA stimulation or DNA virus infection in human cells [14, 15]. In the RNA-sensing pathway, the STING protein functions as a cofactor in the RIG-I-mediated IFN response to RNA viruses [1–3, 11, 16, 17]. Further evidence shows that STING interacts with RIG-I upon viral infection [1, 2]. In addition, STING was identified as a differentially expressed gene induced by the RIG-I agonist 5’pppRNA [18]. In the DNA-sensing pathway, RIG-I can be activated by the B-DNA through an RNA intermediate generated by RNA polymerase III [19, 20].

Innate immunity is essential for protection of the host against DNA pathogens. However, sustained STING activation may lead to autoimmune diseases such as systemic lupus erythematosus (SLE) [21]. Hence, STING activity needs to be tightly regulated. Previous studies have revealed some regulatory mechanisms of STING to avoid excessive activation of innate immune responses. For example, STING is phosphorylated by UNC-51-like kinase (ULK1), leading to STING degradation [22]. NLRC3 can act as a negative regulator of STING-induced innate immune response by impairing the interaction of STING and TBK1 [23]. In addition, RING-finger protein 5 (RNF5) mediates ubiquitination and degradation of STING [16] and TRIM30α acts as a negative-feedback regulator of the innate immune response to intracellular DNA and DNA viruses by promoting degradation of STING in dendritic cells (DCs) [24]. A recent study has shown that the STING protein can be stabilized by sumoylation and the SUMO protease SENP2 may cause degradation of STING after desumoylation [25]. Yet, the STING regulation has not been fully elucidated. In this study, we report that dsDNA induces proteasome-mediated STING degradation in human diploid cells. RIG-I and IL-6 are two negative-feedback regulators of innate immune responses to intracellular DNA by promoting degradation of STING. The study thus provides new insight into STING regulation.

## Materials and methods

### Reagents and cell culture

Bortezomib was purchased from ChemieTek (Indianapolis, IN, USA). The antibodies against STING, RIG-I, phospho-STAT1 (at Tyr701), STAT1, phospho-ULK1 (at Ser556) and ULK1 were purchased from Cell Signaling (Danvers, MA, USA). The antibody against ubiquitin was purchased from Santa Cruz Biotechnology (Santa Cruz, CA). The antibody against β-actin was purchased from Sigma (Milwaukee, WI). The recombinant humanized monoclonal antibody against IL-6 receptor (IL-6R) with the catalog number of HS628 and the monoclonal antibody targeting TNF-α with the catalog number of HS016 were gifted from Zhejiang Hisun Pharmaceutical CO., LTD (Zhejiang, China). Human IL-6 ELISA kit was obtained from R&D systems (Minneapolis, MN). MRC-5 and HEK293 were purchased from ATCC (Rockville, MD). The 2BS and KMB-17 cell lines were established by National Vaccine and Serum Institute in 1973 and Institute of Medical Biology, Chinese Academy of Medical Sciences in 1974, respectively. hUC-MSCs with the catalog number of 20120822C6P5 were gifted anonymously from TuoHua Biotech company (Siping, China), where the cells were isolated from Wharton’s Jelly of a discarded human umbilical cord after normal labor by following the previously described procedures[26], and the donor of the umbilical cord has provided informed consent for the donated tissue to be used in the research. Both HEK293 and all HDCs were cultured in EMEM complete medium supplemented with 10% FBS. hUC-MSCs were cultured in Alpha-MEM complete medium containing 10% FBS.

### Quantitative real-time PCR (qRT-PCR)

Briefly, 1μg of total RNA isolated by Trizol agent (Invitrogen, Carlsbad, CA, U.S.A.) from the treated and control cells were reverse-transcribed by using the SuperScript III First-Strand Synthesis System (Invitrogen). The qRT-PCR assay was performed by using SYBR Premix Ex Taq II kit (Takara, Dalian, China) and the 7500 Fast Real-time PCR system (Applied Biosystem). The following primers were used:

IFN-β forward, 5′-GCTTGGATTCCTACAAAGAAGCA-3′;
IFN-β reverse, 5′-ATAGATGGTCAATGCGGCGTC-3′;
IL-6 forward, 5′-AGTTCCTGCAGAAAAAGGCA-3′;
IL-6 reverse, 5′-AAAGCTGCGCAGAATGAGAT-3′ [14];
IL-8 forward, 5′-CAGCCAAAACTCCACAGTCA-3′;
IL-8 reverse, 5′-TTGGAGAGCACATAAAAACATCT-3′ [27];
IL-1β forward, 5′-CCCAAAGAAGAAGATGGAAAAGC-3′;
IL-1β reverse, 5′-TCTGCTTGAGAGGTGCTGATG-3′ [28];
IL-18 forward, 5′-AGCCAGCCTAGAGGTATGGCTG-3′;
IL-18 reverse, 5′-GATGTTATCAGGAGGATTCATTTCC-3′;
IL-33 forward, 5′-GATGGTAAGATGTTAATGGTAACCC-3′;
IL-33 reverse, 5′-CTGGTCTGGCAGTGGTTTTTC-3′;
STING forward, 5′-AGGACATCCTGGCAGATGCC-3′;
STING reverse, 5′-GGAGAACCTCCTGGGACAGC-3′;
Sendai virus forward, 5′-GGGCGGCATCTGTAGAAATC-3′
Sendai virus reverse, 5′-CGGAAATCACGAGGGATGG-3′
GAPDH forward, 5′-AGAAGGCTGGGGCTCATTTG-3′;
GAPDH reverse, 5′-AGGGGCCATCCACAGTCTTC-3′.

### Western blotting

Whole cell lysates were prepared by using the RIPA buffer containing proteinase inhibitor cocktail (Sigma), which were subject to SDS-PAGE and subsequently transferred onto the nitrocellulose membrane. The membranes were sequentially incubated with appropriate primary antibodies at 4°C overnight, and peroxidase-conjugated secondary antibodies for 2 hours at room temperature. The signals were detected by using ECL Advance Western Blotting Detection Kit (GE Healthcare, Piscataway, NJ).

### Immunoprecipitation

The whole cell lysates were extracted by using RIPA buffer, which were incubated with 1μg of STING antibody at 4°C overnight, followed by incubating with protein A/G agarose at 4°C for 1 h. After washing three times with RIPA buffer at room temperature, the agarose-bound proteins were analyzed by western blotting using an ubiquitin antibody.

### siRNA and plasmid transfection

After cells reach approximately 90% confluency in a 35-mm culture dish, 75 pmol of target-specific siRNA or negative control siRNA with 7.5 μl of RNAiMAX in 250 μl of Opti-MEM were transfected according to the manufacturer’s instructions (Invitrogen). For plasmid transfection, 200 μl of transfection mixture containing 0.01–0.5 μg plasmid DNA and 5μl of Lipofectamine 2000 (Lipo 2000) (Invitrogen) were applied. The siRNA knockdown efficiency was determined by western blotting. Each siRNA knockdown experiment was repeated at least twice. The sequences for siRNA oligonucleotides targeting STING protein are the following: siSTING-1, 5′-CCGGATTCGAACTTACAATTT-3′; siSTING-2, 5′-CTGGCATGGTCATATTACA-3′; For the silencing of RIG-I, the siRNA were chosen from published paper [29] and the sequence for siRIG-I is 5′- CCACAACACUAGUAAACAA-3′. The siRNA control was purchased from Invitrogen.

### Luciferase assay for detecting rTV-Fluc virus replication

The rTV-Fluc was a recombinant and replication-attenuated TianTan Vaccinia virus carrying a firefly luciferase gene of Photinus pyralis [30]. To test rTV-Fluc virus replication, the MRC-5 cells were infected with rTV-Fluc (MOI = 0.01) after 24 hours of siRNA or plasmid transfection. After 24 h after infection, the luciferase activity was measured by using the Glomax Luminometer and Bright-Glo^™^ Luciferase Assay System (Promega, Madison, WI, USA). The test was performed in triplicates for each sample and each test was repeated at least twice.

### Data analysis

The Data from qRT-PCR and luciferase assay were expressed as means±SEM of at least three separate experiments. Comparison between group means was assessed using one-way analysis of variance with Newman-Keuls posttest in GraphPad Prism 6 Software (San Diego, CA). The difference with p<0.05 was considered statistically significant. The western blotting results were quantified by using Gel-pro Analyzer software.

## Results

### STING serves as a critical mediator for the plasmid DNA-induced IFN-β response and the inhibition of virus replication

**Fig 1:**
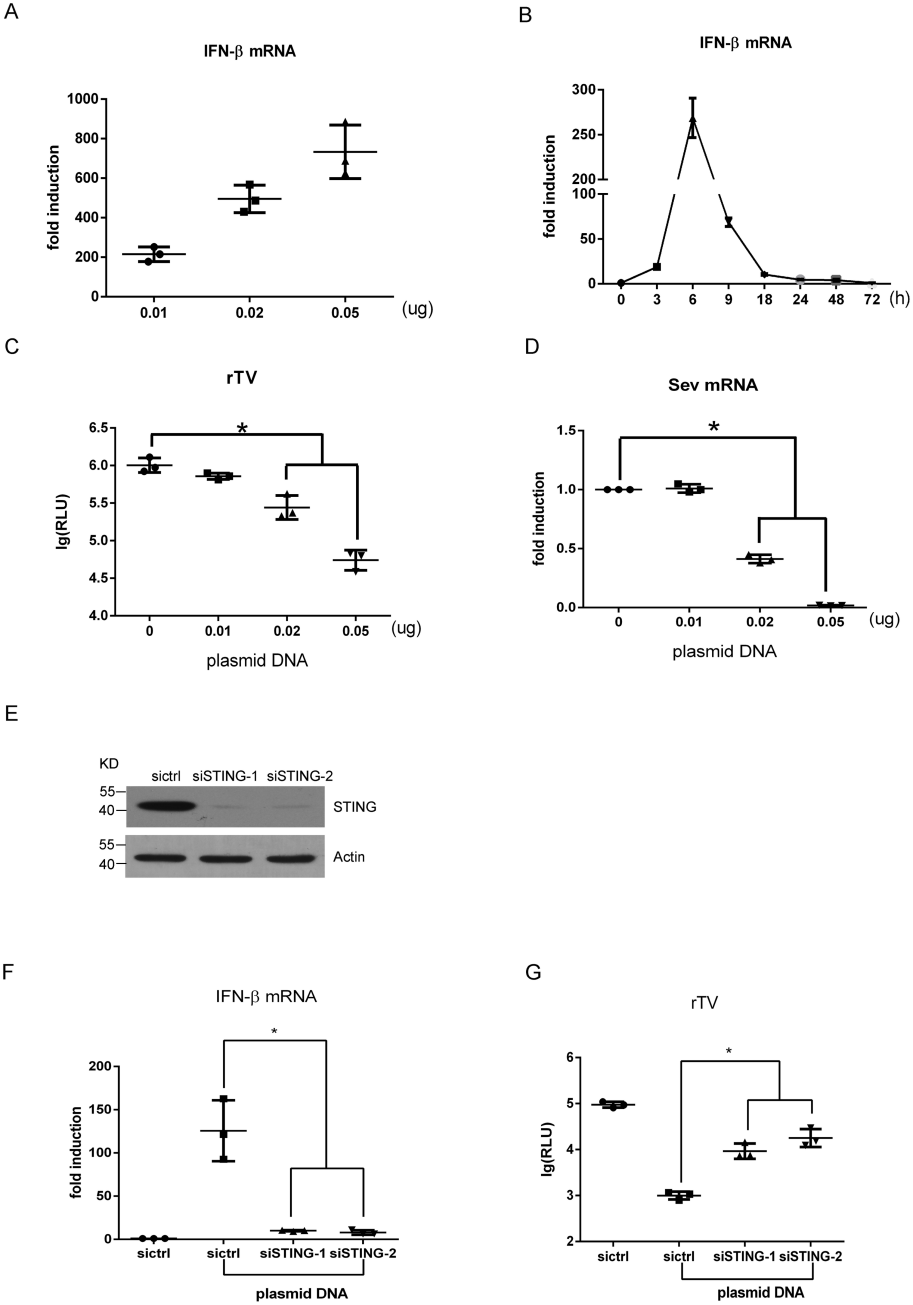
STING serves as a major mediator for the plasmid DNA-induced IFN-β response. (A) Analysis of the dose effect on IFN-β production induced by DNA transfection. MRC-5 cells were transfected with pcDNA3.1 with indicated amounts for 6 hours. IFN-β mRNA was analyzed by qRT-PCR. (B) Time course assessment of IFN-β induction by pcDNA3.1 (0.05μg). (C, D) After 24 h transfection of MRC-5 cells with pcDNA3.1, cells were infected with rTV-Fluc (C) or Sendai virus (D) for another 24 hours before a luciferase report assay for rTV-Fluc (C) and qRT-PCR for Sendai virus (D). (E) Western blotting showing STING knockdown by siRNA after 72 h transfection in MRC-5 cells. (F) qRT-PCR assessment of IFN-β after STING was knockdown in MRC-5 cells that were transfected with pcDNA3.1(0.2μg). (G) A luciferase report assay showing rTV-Fluc virus replication after STING knockdown. Data are presented as mean ± SD (n = 3). Asterisk (*), P <0.05.

Double-stranded DNA (dsDNA) that are derived from viruses and bacteria or generated from the incompletely digested cytosolic DNA of the host cells are important immunity activators [31]. To investigate the molecular mechanism underlying the innate immune response represented by the IFN-β production, we assessed the effect of pcDNA3.1 plasmid DNA on IFN-β production and viral replication in MRC-5, a human diploid cell line has been commonly used in production of viral vaccines. As expected, we found that the DNA transfection induced a significant increase in IFN-β expression in a dose- and time-dependent manner (Fig 1A and 1B) and a remarkable reduction in the replication of both rTV and Sendai virus in a dose-dependent manner (Fig 1C and 1D), suggesting that the dsDNA induced an IFN-β response and subsequent antiviral activity in MRC-5 cells.

STING is a critical mediator of the DNA-triggered type I IFN response [1–4]. However, such studies have been mainly focused on non-HDC cell lines such as HEK293 cells [2], mouse embryonic fibroblasts (MEFs) [11], and immune cells such as dendritic cells and macrophages [11]. We were particularly interested in HDC cells such as the MRC-5 cell line because they are commonly used in the production of viral vaccines. Understanding the innate immune signaling pathway in MRC-5 cells may help develop more efficient strategies to promote vaccine production. To determine whether the STING protein serves as a mediator of the dsDNA-induced IFN-β response in MRC-5 cells, we silenced STING expression before transfecting MRC-5 cells with plasmid DNA. Western blotting showed that STING protein was remarkably depleted by the STING-specific siRNA (siSTING) (Fig 1E). As expected, the STING knockdown nearly abolished the plasmid DNA-induced induction of IFN-β (Fig 1F), and the inhibition of the virus replication by plasmid DNA was also greatly reversed by the STING depletion (Fig 1G). These data indicate that STING plays an important role in mediating plasmid DNA-induced IFN-β response and the subsequent inhibition of virus replication in HDC cells.

### Plasmid DNA induces STING protein reduction in MRC-5 cells

**Fig 2:**
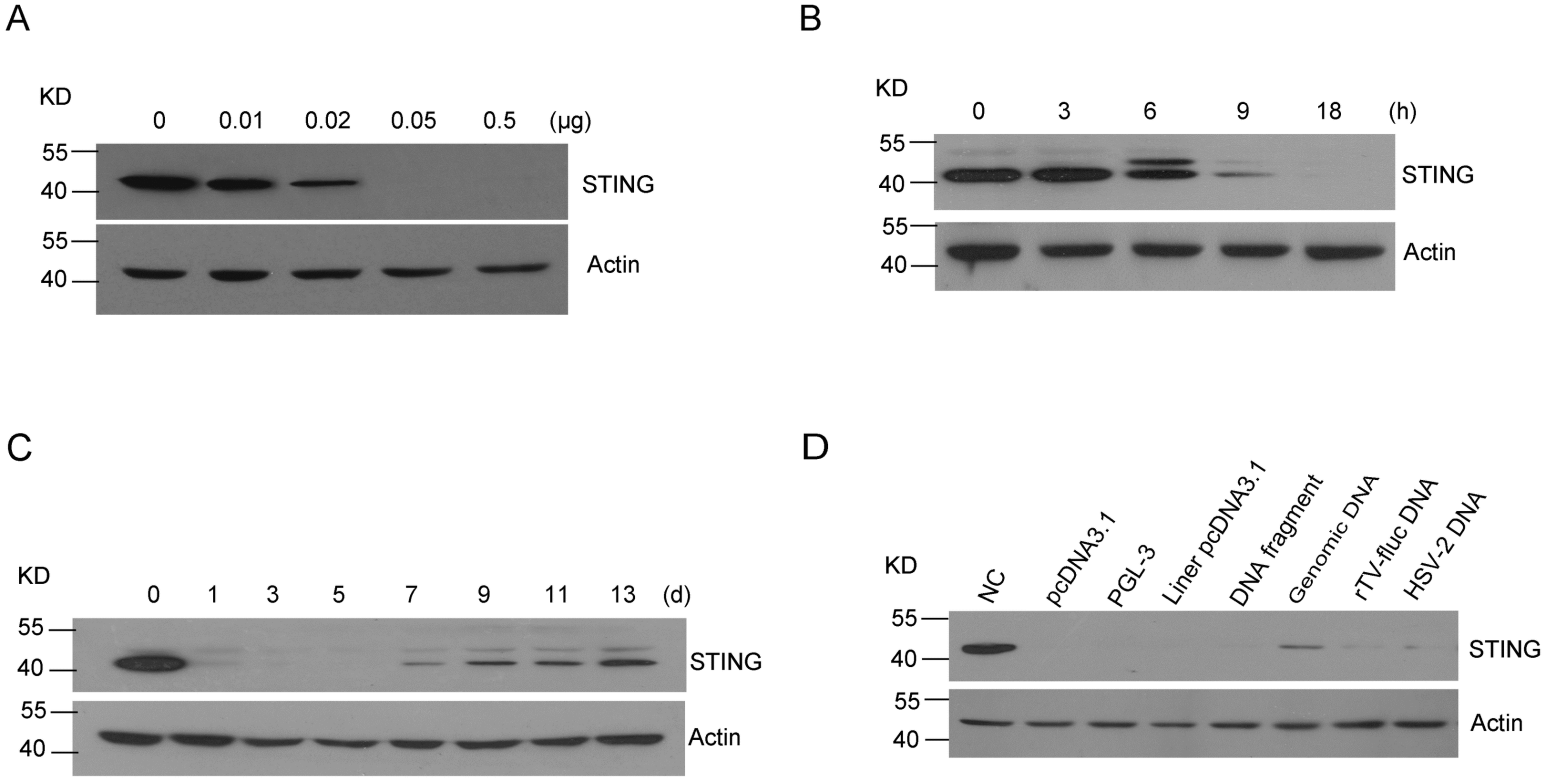
The plasmid DNA induces STING protein reduction in MRC-5 cells. (A) After 48 h transfection with different concentrations of pcDNA3.1, the MRC-5 cell lysates were analyzed by immunoblotting with anti-STING. (B, C) Time course assessment of STING expression induced by DNA. (D) Western blotting assessment of STING after 48 h transfection of MRC-5 cells with equal amount of circular plasmid pcDNA3.1, plasmid vector PGL-3, liner plasmid pcDNA3.1, 2 kb PCR DNA fragment amplified from pcDNA3.1, genomic DNA derived from MRC-5 cells, DNA virus (rTv-Fluc and HSV-2).

Considering the importance of STING in mediating the dsDNA-induced responses, we next assessed the STING protein expression using Western blotting after DNA transfection in MRC-5 cells. Strikingly, we found that the DNA transfection induced a significant dose-dependent reduction of STING protein, which became remarkably reduced when greater than 0.05 μg plasmid DNA was used in the transfection (Fig 2A).

To further characterize DNA-induced STING reduction, we performed a time course experiment and found that STING protein was greatly reduced at 9 h and was nearly undetectable 18 h after transfection with 0.05 μg plasmid DNA (Fig 2B). In addition, 6 h after the DNA transfection, we observed the appearance of an extra STING band with a slightly higher molecular weight than the original band, which was likely due to an increase in phosphorylation modification [22]. STING reduction was maintained for 5–6 days after the transfection mixture was replaced with regular complete medium at 72 h and it started to recover at day 7 after transfection (Fig 2C).

Besides the circular pcDNA3.1 plasmid DNA, we also tested the effect of other double- stranded DNAs, such as linearized pcDNA3.1 produced by digestion of the circular pcDNA3.1, pGL-3 plasmid DNA, a 2kb PCR fragment amplified from pcDNA3.1 plasmid DNA, the ultrasonic-sheared genomic DNA from MRC-5 cells, and the DNA isolated from rTV-Fluc and HSV-2, in comparison with the circular pcDNA3.1 DNA. Equal amounts of molecules of all types of dsDNAs were used for transfection. The results showed that all types of dsDNAs caused a similar degree of STING reduction (Fig 2D).

### Proteasome-mediated degradation plays a role in STING reduction

**Fig 3:**
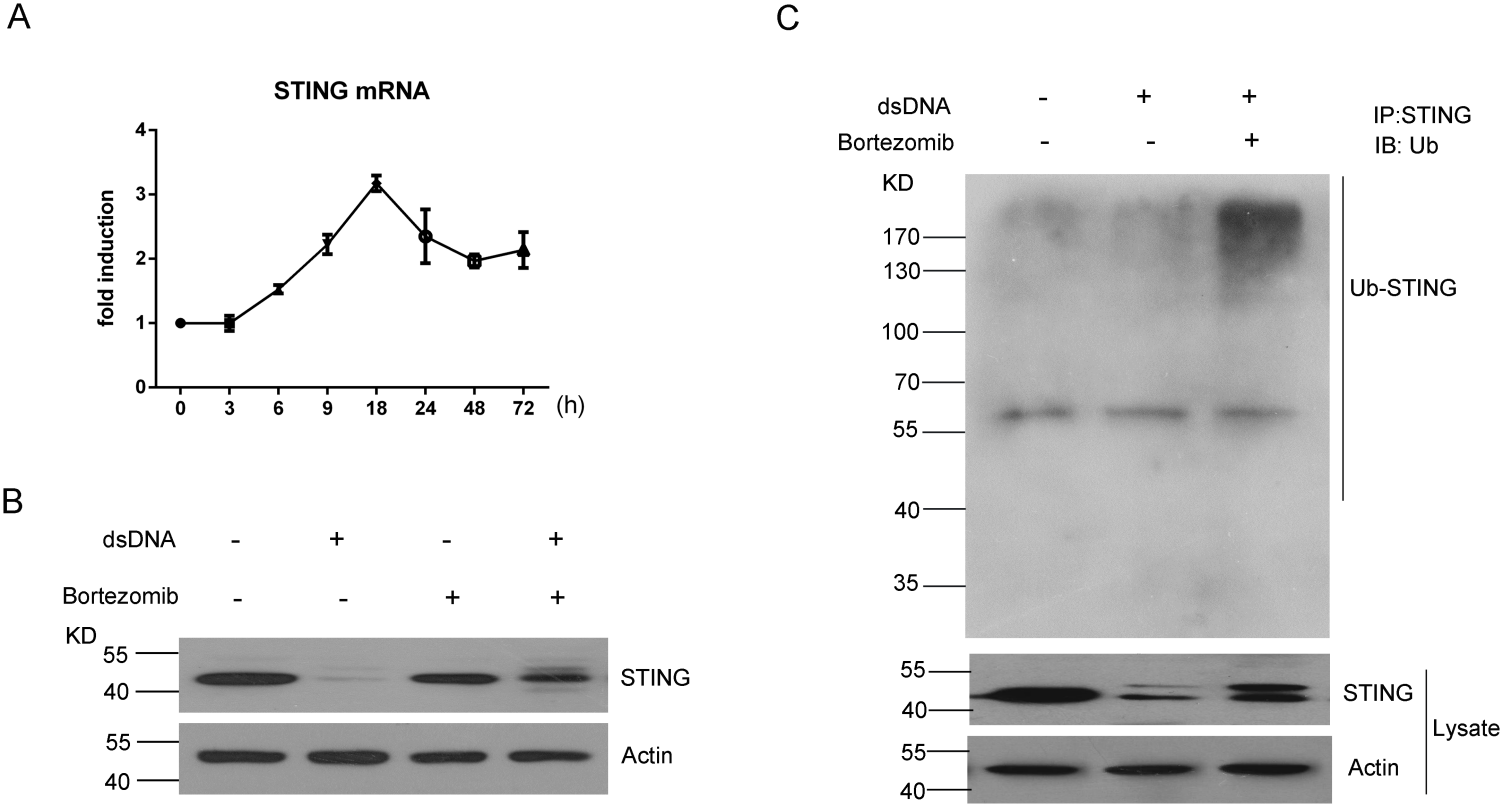
Proteasome-mediated degradation plays a role in STING reduction. (A) Time course assessment of STING mRNA expression after MRC-5 cells were transfected with pcDNA3.1 (0.05μg). (B) Western blotting assessment of STING after MRC-5 cells were transfected with pcDNA3.1 (0.05μg) with treatment of Bortezomib (20 nM) or DMSO for 18h. (C)Western blotting assessment of immunoprecipitated STING with anti-ubiquitin antibody.

To investigate the molecular mechanism responsible for the dsDNA-induced STING reduction, we examined the STING mRNA expression in a time course experiment. Interestingly, a 2 to 3-fold increase in STING mRNA was observed after plasmid DNA transfection (Fig 3A), suggesting that post-translational modification might account for the STING protein reduction.

Since proteasome-mediated protein degradation contributes to the degradation of vast majority of proteins outside of lysosomes, we assessed the role of the proteasome in the regulation of STING by using the proteasome inhibitor Bortezomib. We found that treatment with 20 nM Bortezomib greatly reversed the STING protein reduction, suggesting that the STING underwent a proteasome-mediated degradation in dsDNA-transfected MRC-5 cells (Fig 3B). Furthermore, we used an immunoprecipitation assay to pull down the STING protein from the MRC-5 cell lysates after plasmid transfection in the presence or absence of Bortezomib. Precipitated STING by the specific antibody was probed by anti-ubiquitin antibody. The results showed that Bortezomib treatment led to a long smear band representing the accumulated polyubiquitin-conjugated STING proteins, supporting that the proteasome-mediated degradation was involved in the reduction of STING protein (Fig 3C).

### RIG-I is involved in regulating the dsDNA-induced STING degradation

**Fig 4:**
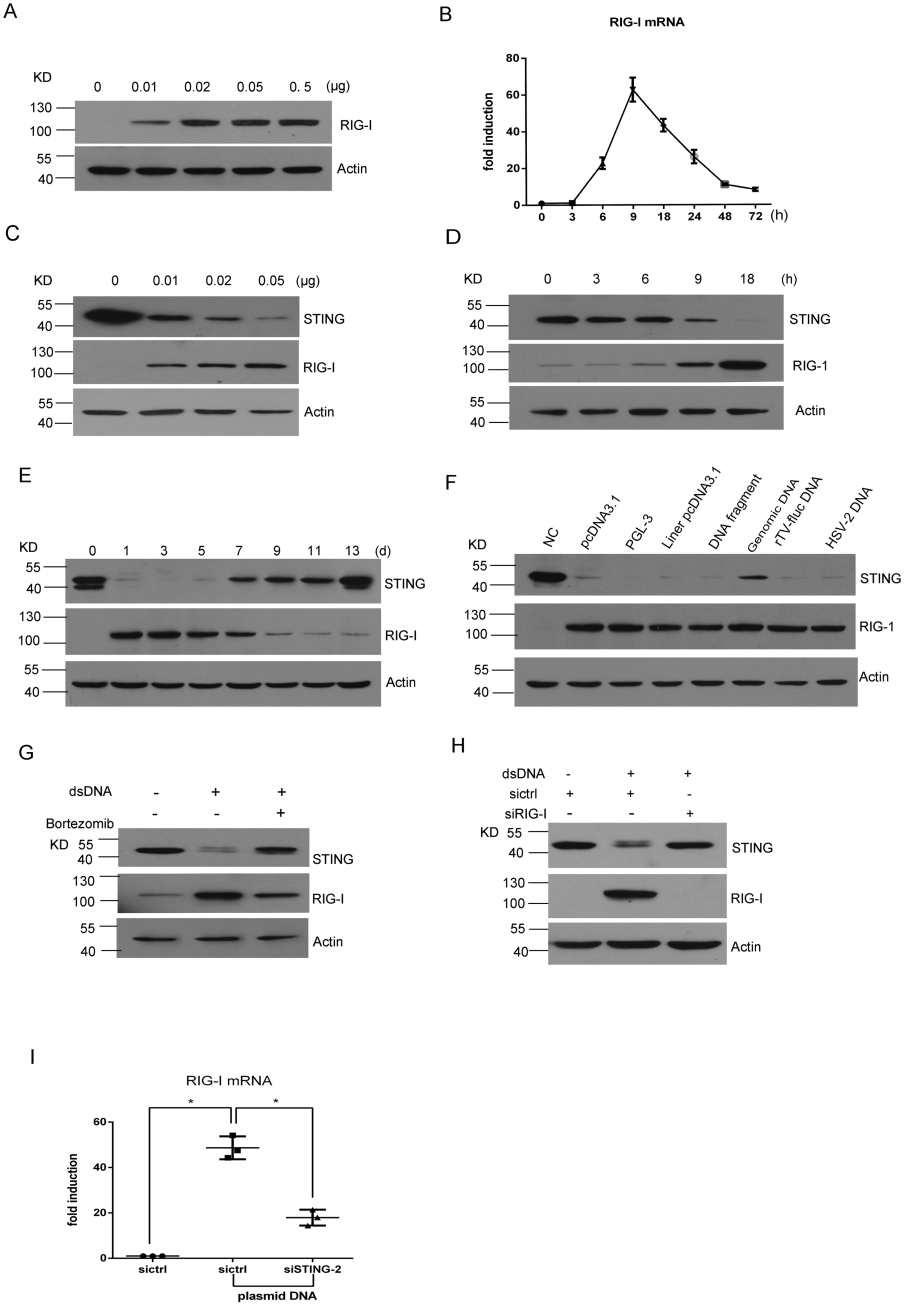
RIG-I is involved in regulating the DNA-induced STING degradation. (A) Western blotting analysis of RIG-I expression after MRC-5 cells were transfected with pcDNA3.1. (B) Time course assessment of RIG-I mRNA after MRC-5 cells were transfected with pcDNA3.1 (0.05μg) for the indicated time points. (C-G) Western blotting shows a reverse correlation between STING and RIG-I under different conditions: dose (C), time (D, E), DNA types (F), and Bortezomib treatment (G). (H) Silencing RIG-I partially reverses the STING degradation. (I) qRT-PCR assessment of RIG-I expression after STING knockdown in MRC-5 cells. Asterisk (*), P <0.05.

RIG-I, a well-known cytosolic RNA receptor, is capable of detecting cytosolic RNA and mediating RNA-associated innate immune responses [12, 13]. In addition, several reports have shown that RIG-I is involved in DNA sensing pathway [14, 15]. We therefore examined the role of RIG-I in the regulation of STING expression in MRC-5 cells after dsDNA transfection. We found that the dsDNA transfection dramatically induced a dose-dependent increase of RIG-I expression (Fig 4A). qRT-PCR showed that RIG-I mRNA was detectable at 6 h, reached peak levels at 9 h, then declined within 48 h although still detectable at 72 h (Fig 4B).

Interestingly, the increase of RIG-I was inversely correlated with reduction of STING in both time- and dose-dependent manners (Fig 4C–4E). Similar to the circular pcDNA3.1 DNA, different types of DNA were also able to induce RIG-I expression (Fig 4F). The inverse correlation was even observed in the cells treated with Bortezomib, where increased STING expression was accompanied by decreased RIG-I (Fig 4G), suggesting that RIG-I was likely involved in STING degradation. To test this hypothesis, we co-transfected MRC-5 cells with specific siRNA against RIG-I and plasmid DNA. Two sequences were designed for silencing RIG-I protein expression according to the published paper [29], and we chose one sequence to test the involvement of RIG-I in the regulation of STING degradation based on the comparison of the efficiency in reducing RIG-I protein expression (data not shown). Western blotting showed that silencing of RIG-I partially reversed STING reduction (Fig 4H), suggesting that RIG-I was involved in the regulation of STING degradation.

To further delineate the relationship of STING and RIG-I, we transfected MRC-5 cells with siSTING together with plasmid DNA and assessed expression of RIG-I. The results showed that the increase of RIG-I was significantly blocked by STING knockdown (Fig 4I), suggesting that the increase of RIG-I was a downstream event of STING activation and implicated RIG-I mediating a negative feedback regulation of STING expression.

### IL-6 and RIG-I contribute additively to the DNA-induced STING degradation

**Fig 5:**
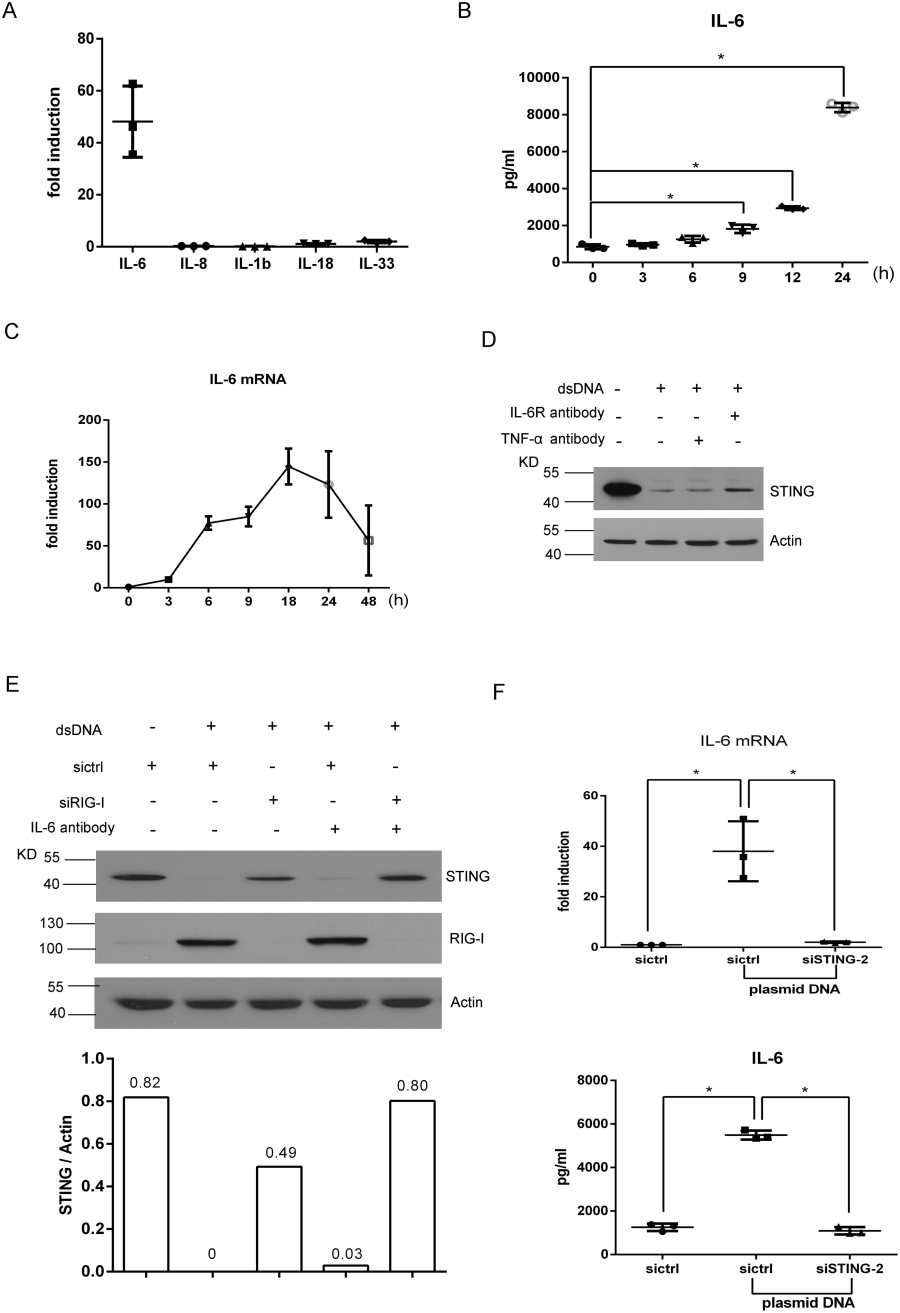
IL-6 and RIG-I contribute additively to the DNA-induced STING degradation. (A) qRT-PCR assessment of expression of indicated interleukins after MRC-5 cells were transfected with pcDNA3.1 (0.05 μg) for 9 hours. (B) ELISA analysis of IL-6 protein levels in the supernatants of cells at different time points. (C) qRT-PCR analysis of IL-6 mRNA at different time points. (D) Western blotting assessment of STING expression after MRC-5 cells were treated with IL-6R antibody with pcDNA3.1 transfection. (E) Western blotting assessment of STING expression after application of IL-6R antibody and siRIG-I. (F) qRT-PCR (up) and ELISA analysis (down) of IL-6 expression after STING knockdown. Asterisk (*), P <0.05.

Given that RIG-I silencing only partially reversed DNA-induced STING degradation, we speculated that other molecules might be also involved in the negative feedback regulation of STING expression. Interleukins are important cytokines upregulated in response to DNA stimulation and participate in the regulation of immune responses. To determine whether interleukins were involved in the regulation of STING degradation, we tested the expression of IL-6, IL-8, IL-1b, IL-18 or IL-33 by qRT-PCR after 9 h of DNA transfection since the greatest STING reduction was appreciated at this time point (Fig 2B). We found that, among all interleukins tested, IL-6 expression showed more than 40-fold increase, whereas others showed less than 3-fold increase (Fig 5A). Then we measured the IL-6 protein levels in the supernatants of cells by a time course experiment. ELISA results showed that the amount of IL-6 protein was gradually increased after 9 h after plasmid transfection (Fig 5B). The qRT-PCR results showed that the IL-6 induction appeared temporally similar to the induction of IFN-β and RIG-I (Fig 5C).

To test whether IL-6 was also involved in regulating STING degradation, we used an IL-6R antibody to block its effects and a TNF-α neutralizing antibody as an isotype control, which is not expressed in MRC-5 cells before or after transfection of pcDNA3.1 (data not shown). We found that the treatment with IL-6R blocking antibody, but not the isotype control, partially reversed the dsDNA-induced STING degradation, indicating that IL-6 was involved in the regulation of STING degradation (Fig 5D). More importantly, the combination of IL-6R antibody and RIG-I knockdown resulted in a clearly additive effect in reversing STING reduction (Fig 5E), demonstrating that both RIG-I and IL-6 contributed to the DNA-induced STING degradation. Since the induced expression of both IL-6 and RIG-I appear to be downstream events of STING (Figs 5F and 4I), these results support the hypothesis that additive effects from IL-6 and RIG-I on the STING degradation resulted from a negative feedback mechanism.

### RIG-I or IL-6 promotes STING degradation by activating ULK1 in MRC-5 cells

**Fig 6:**
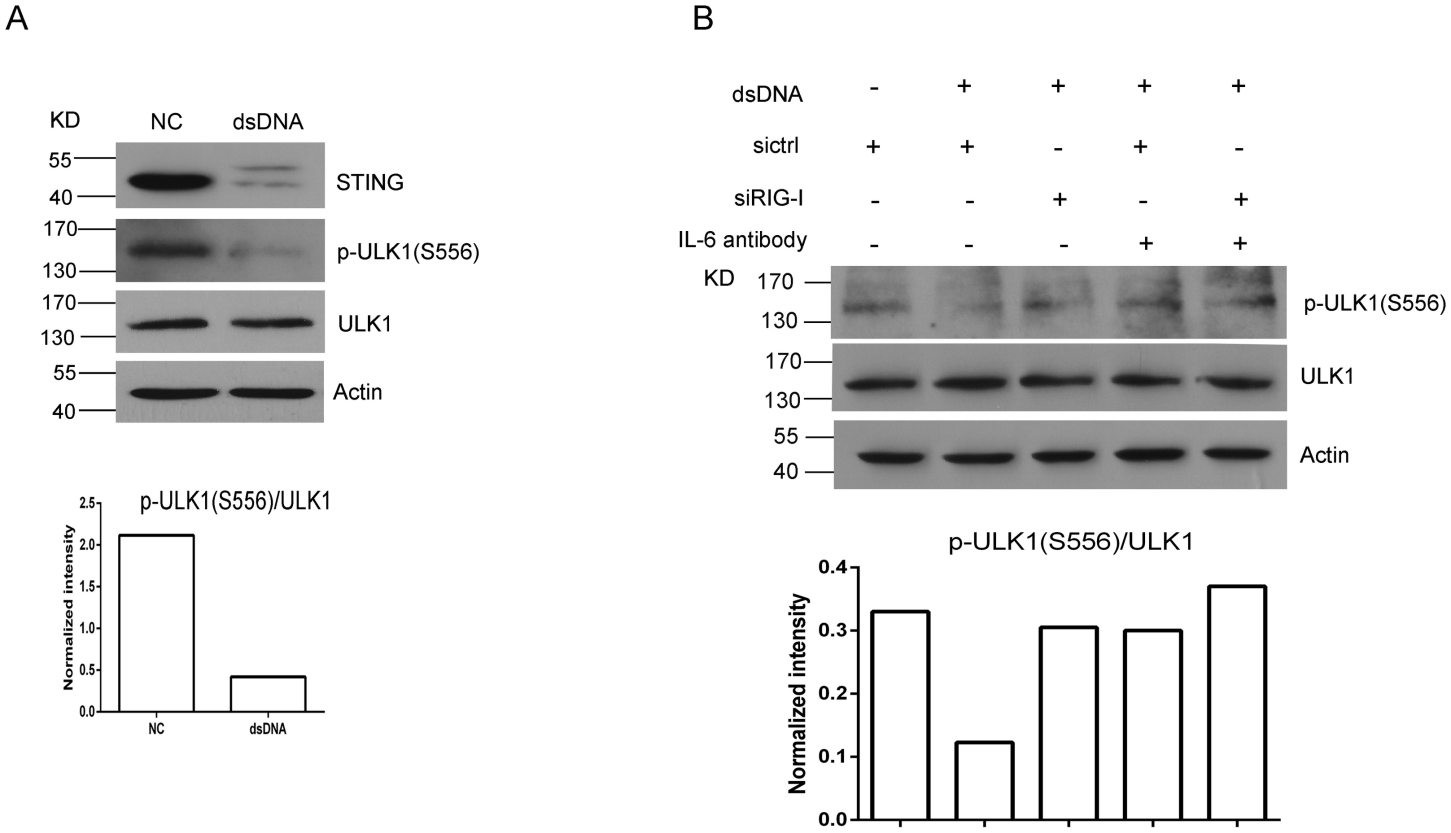
RIG-I or IL-6 promotes STING degradation by activating ULK1 in MRC-5 cells. (A) Western blotting assessment of the levels of ULK1 S556 phosphorylation triggered by dsDNA. (B) Western blotting assessment of the ULK1 S556 phosphorylation in MRC-5 cells with RIG-I knockdown or IL-6R antibody treatment.

A previous study has revealed that ULK1 is responsible for STING degradation in hTERT-BJ1 cells in response to exogenous dsDNA [22]. The ULK1 activity is inhibited by AMP activated protein kinase (AMPK), which phosphorylates S556 of ULK1 [22]. When AMPK dissociates from ULK1, leading to the dephosphorylation of S556, thereafter ULK1 is activated. To identify the mechanisms of dsDNA-induced STING degradation in MRC-5 cells, we assessed ULK1 S556 phosphorylation after dsDNA transfection. The results showed that the levels of ULK1 S556 phosphorylation were remarkably decreased (Fig 6A), suggesting that ULK1 is involved in dsDNA-induced STING degradation in MRC-5 cells. To test whether RIG-I or IL-6 promotes STING degradation through activation of ULK1, we tested the S556 phosphorylation of ULK1 in MRC-5 cells after siRIG-I transfection or/and IL-6R antibody treatment with dsDNA transfection. The western blotting results showed that RIG-I knockdown or IL-6R antibody treatment effectively reversed the dephosphorylation of ULK1 triggered by dsDNA (Fig 6B), suggesting that RIG-I or IL-6 promotes STING degradation by activating ULK1.

### STING degradation occurs in HDCs but not in HEK293

**Fig 7:**
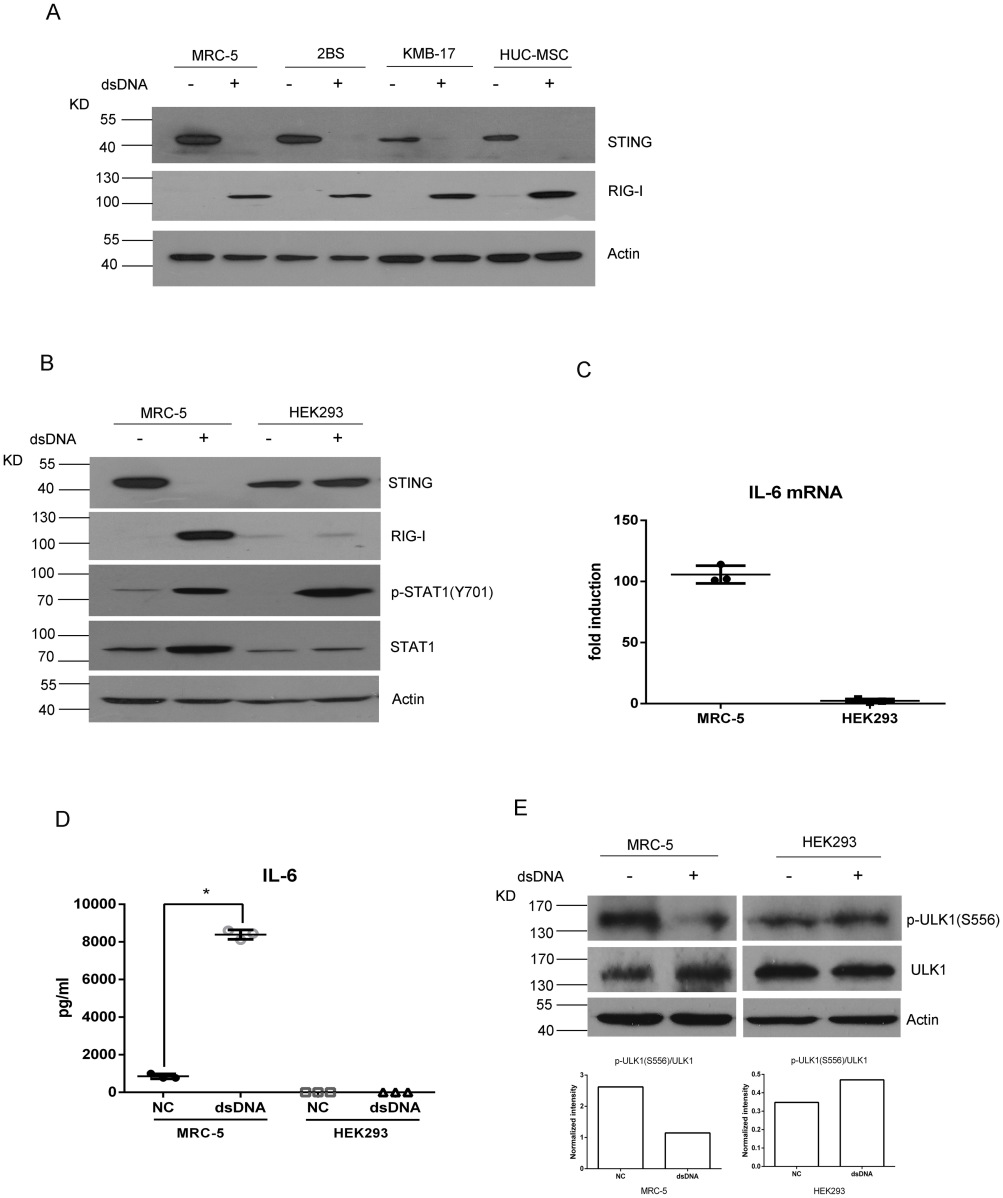
STING degradation occurs in HDCs but not in HEK293. (A) Western blotting assessment of STING and RIG-I in MRC-5, 2BS, KMB17 and hUC-MSC cells after transfection with 0.5 μg of pcDNA3.1 for 24 h. (B) MRC-5 and HEK293 cells were transfected with 0.5 μg of pcDNA3.1 for 24 hours. The indicated molecules were assessed by western blotting. (C, D) qRT-PCR assessment of mRNA levels (C) and ELISA assessment of protein levels (D) of IL-6 in MRC-5 and HEK293 cells after transfection of pcDNA3.1. (E) ULK1 S556 phosphorylation was assessed by western blotting in MRC-5 and HEK293 cells. Asterisk (*), P <0.05.

To investigate whether other human diploid cells also undergo STING degradation induced by dsDNA transfection, we tested two other HDCs (2BS and KMB17) and a human umbilical cord mesenchymal stem cell line (hUC-MSCs). We found that the STING protein in all HDCs and hUC-MSCs underwent a similar degradation after dsDNA transfection, accompanying with RIG-I induction (Fig 7A). However, in HEK293 cells, changes in STING and RIG-I were not significant at the protein level after DNA transfection, although the increased expression of phosphorylated STAT1 indicates that HEK293 was able to respond to the transfected DNA (Fig 7B). Meanwhile, compared with MRC-5 cells, the mRNA level of IL-6 was only slightly increased (Fig 7C), and the IL-6 protein was undetectable after DNA transfection in HEK293 cells (Fig 7D), suggesting that the STING degradation is a common phenomenon in HDCs but not in non-HDC HEK293 cells. The difference in RIG-I and IL-6 levels between MRC-5 and HEK293 cells further supports that both are involved in mediating STING degradation in HDCs.

To explain why normal diploid cells degraded STING, whereas HEK293 was unable to do so, we tested the phosphorylation of ULK1 in both MRC-5 and HEK293 cells. Western blotting showed that, unlike MRC-5 cells, the loss of ULK1 S556 phosphorylation was not observed in HEK293 cells after dsDNA transfection (Fig 7E), suggesting that the low levels of RIG-I and IL-6 are insufficient to activate ULK1, and thus ULK1 remains in an inactive state, which is unable to induce STING degradation in HEK293 cells.

## Discussion

Cytosolic DNA from either intrinsic or extrinsic origins is a potent immune activator and is able to evoke a robust type I IFN and innate immune responses [32]. The production of type I IFNs induced by cytosolic DNA depends on STING [1, 3, 11] and if not contained the STING activity, the persistent production of the pro-inflammatory molecules induced by DNA could stimulate uncontrolled immune responses, resulting in autoimmune diseases. Therefore, it is reasonable to hypothesize that a mechanism restricting the effect of sustained STING activation induced by dsDNA must exist in normal cells. Based on the literature reports, we learned that the STING activity is negatively regulated by multiple mechanisms including ULK1-mediated phosphorylation [22], impediment of its interaction with TBK1 by NLRC3 [23], RNF5-mediated ubiquitination and degradation [16], and accelerated degradation by TRIM30α [24]. Interestingly, our present study provides a new mechanism for controlling STING activity by its downstream molecules RIG-I and IL-6, which negatively regulate the stability of STING protein.

Here we demonstrated that RIG-I was involved in dsDNA signaling pathway in HDC cells, which was supported by the evidence of dose-dependent increase of RIG-I expression after introduction of dsDNA into MRC-5 cells. However, the role of RIG-I may be different from previous findings [14, 15]. Using RNAi knockdown, we confirmed that STING was essential for the production of RIG-I as well as IL-6 and and knockdown of RIG-I or blockage of IL-6 signaling pathway partially reversed the degradation of STING protein induced by the dsDNA. Therefore, we conclude that RIG-I and IL-6 are downstream components of the STING pathway, and the stability of STING regulated by RIG-I and IL-6 reflects a negative feedback mechanism for limiting the dsDNA-induced uncontrolled innate immune response in HDC cells. In our experiment, we also tested the effect of RIG-I or IL-6 alone on the degradation of STING protein by WB in MRC-5 cells, and neither RIG-I induced by type I IFN treatment nor IL-6 alone can induce STING degradation (data not shown). Putting all findings together, it can be concluded that the STING degradation was caused by a combined effect of the increased expression of RIG-I and IL-6 with each one playing a limited role. In addition, we confirmed that the STING degradation induced by RIG-I and IL-6 was a consequence of ULK1 activation, which was caused by activation of AMPK and sequential dephosphorylation of ULK1 at S566 [22]. However, the mechanism for RIG-I and IL-6 regulating the AMPK-ULK1 axis needs to be further studied.

The human diploid cell lines such as MRC-5 and WI-38 have been widely used to produce a variety of vaccines against a variety of viruses including Hepatitis A, Rubella, Varicella, Zoster, Adenovirus and Rabies. Although these cells had been well characterized, further understanding of the cellular features of these cells may help produce safer vaccines. In this study, we found that STING degradation is common in HDCs. However, in HEK293 cells, STING degradation and the increase of RIG-I and IL-6, as well as the dephosphorylation of ULK1 were not greatly appreciated after transfection with dsDNA. HEK293 cells were generated by transformation of normal human embryonic kidney cells with sheared adenovirus 5 DNA. HEK293 cells have a very complex karyotype, containing less than three times the number of chromosomes of a normal human diploid cell. In addition, different from normal diploid cells, HEK293 cells have the potential for an infinite expansion. Based on the different characteristics of HEK293 and HDC cells, we speculate that the negative feedback regulation mechanism, which exists in normal HDCs, might have been lost in HEK293 cells during the process of transformation or passage.

We also found that different types of dsDNA tested in this study were able to induce a similar STING degradation, indicating that STING degradation is a common feature of MRC-5 cells in response to dsDNA transfection. Our data may have other important implications in basic biology. Introduction of plasmids into cells for studying biological functions is a commonly used method. Our study shows that dsDNA may induce a cellular defense mechanism, resulting in subsequent activation of type I interferons and proteasome degradation. Thus, caution should be taken when interpreting data obtained from such experiments.

In summary, our study has proposed that STING degradation in HDCs is due to a negative feedback triggered by the exogenous DNA in order to avoid excessive innate immune response. RIG-I and IL-6 are two major regulators of STING stability. In addition, RIG-I and IL-6 regulate STING degradation by activating ULK1. Secondly, we have shown that the regulatory mechanism of STING in HEK293, which has been widely used in the study of STING signaling pathway [2, 3], differs from that in HDCs.
